# *CBS* promoter hypermethylation increases the risk of hypertension and stroke

**DOI:** 10.6061/clinics/2019/e630

**Published:** 2019-03-14

**Authors:** Changyi Wang, Guodong Xu, Qi Wen, Xiaolin Peng, Hongen Chen, Jingwen Zhang, Shan Xu, Chunhui Zhang, Min Zhang, Jianping Ma, Zhaohui Hui, Guifu Wu, Min Ma

**Affiliations:** I Integrated Chinese and Western Medicine Postdoctoral research station, Jinan University, Guangzhou, China.; II College of Traditional Chinese Medicine of Jinan University, Institute of Integrated Traditional Chinese and Western Medicine of Jinan University, Guangzhou, China.; Jinan University, Jinan University, Institute of Integrated Traditional Chinese and Western Medicine, Guangzhou, China; III Department of Cardiology. The Eighth Affiliated Hospital of Sun Yat-sen University. Shenzhen, China.; IV Department of Non-communicable Disease Prevention and Control, Shenzhen Nanshan Center for Chronic Disease Control, Shenzhen, China.; V Department of Preventive Medicine, School of Medicine, Ningbo University, Ningbo, China.; VI Shenzhen Xili People's Hospital, Shenzhen, China.

**Keywords:** CBS, Hypermethylation, Hypertension, Stroke, Homocysteine

## Abstract

**OBJECTIVES:**

Cystathionine β-synthase is a major enzyme in the metabolism of plasma homocysteine. Hyperhomocysteinemia is positively associated with hypertension and stroke. The present study was performed to examine the possible effects of Cystathionine β-synthase promoter methylation on the development of hypertension and stroke.

**METHODS:**

Using quantitative methylation-specific PCR, we determined the Cystathionine β-synthase methylation levels in 218 healthy individuals and 132 and 243 age- and gender-matched stroke and hypertensive patients, respectively. The relative changes in Cystathionine β-synthase promoter methylation were analyzed using the 2^–^ΔΔ^Ct^ method. The percent of the methylated reference of Cystathionine β-synthase was used to represent the Cystathionine β-synthase promoter methylation levels.

**RESULTS:**

In this study, the Cystathionine β-synthase promoter methylation levels of hypertensive and stroke participants were both higher than that of the healthy individuals (median percentages of the methylated reference were 50.61%, 38.05% and 30.53%, respectively, all *p*<0.001). Multivariable analysis showed that Cystathionine β-synthase promoter hypermethylation increased the risk of hypertension [odds ratio, OR (95% confidence interval, CI)=1.035 (1.025–1.045)] and stroke [OR (95% CI)=1.015 (1.003–1.028)]. The area under the curve of Cystathionine β-synthase promoter methylation was 0.844 (95% CI: 0.796–0.892) in male patients with hypertension and 0.722 (95% CI: 0.653–0.799) in male patients with stroke.

**CONCLUSION:**

Cystathionine β-synthase promoter hypermethylation increases the risk of hypertension and stroke, especially in male patients.

## INTRODUCTION

Hypertension is the leading preventable risk factor for premature death worldwide (1). Stroke is the third leading risk factor for death in most Western developed countries (2), but it has become the most common cause of death in China along with the development in social economy (3). After adjustment for age, the mortality of stroke was approximately 115 per 100,000 person-years in China (4).

Hypertension and stroke are both multifactorial disorders that are affected by environmental factors, genetic alterations and gene-environment interactions (5,6). Epigenetic modifications of the genome were also shown to be involved in the pathophysiological course of hypertension and stroke (7). DNA methylation is one of the most common epigenetic modifications and is primarily involved in control of gene expression (8). Promoter DNA that is highly methylated may cause transcriptional silencing of genes, while DNA with little methylation may result in the promotion of gene transcription (9). Abnormal methylation levels of *ADD1* (10), *ACE* (11), and *AGTR1* (12) were associated with the pathogenesis of hypertension. Aberrant DNA methylation levels of *LINE-1* (13), *AS* (14) and *ABCB1* (15) have been associated with the development of stroke.

Cystathionine β-synthase (*CBS*) is a cytosolic homotetramer composed of 63 kDa subunits, and as a key enzyme involved in the metabolism of plasma homocysteine (Hcy), it mainly converts Hcy and serine to cysteine (16). A study of the American population demonstrated that the *CBS* gene was related to abnormal Hcy levels (17). Low levels of the *CBS* gene lead to hyperhomocysteinemia, which was also discovered in earlier studies (18, 19), indicating its effects on hypertension and stroke. However, a direct association between *CBS* methylation and hypertension and stroke remains unclear.

This matched case-control study aimed to investigate the relationship between *CBS* methylation and the risk of hypertension and stroke in a Chinese population.

## MATERIALS AND METHODS

### Subjects

This matched case-control study consisted of 132 stroke patients, 218 healthy individuals, and 243 matched hypertensive patients from the Hypertension Management Information System in Community Health Service Centers (CHSCs) in the Nanshan district, Shenzhen, Guangdong Province, China. All participants were local residents living in Shenzhen for more than six months and were registered in CHSCs. The hypertensive patients were diagnosed based on a systolic blood pressure (SBP) ≥140 mmHg and/or diastolic blood pressure (DBP) ≥90 mmHg or a self-reported history of using antihypertensive medications (20). Two neurologists independently diagnosed the stroke (ischemic, intracerebral and subarachnoid hemorrhagic) according to WHO diagnostic criteria (21). Those who had a history of secondary hypertension, malignant tumors, liver and kidney failure, or pregnancy were excluded, and patients with a history of using vitamin B6, vitamin B12, or folic acid were also eliminated. All participants signed informed consent. The Research Ethical Committee of the Nanshan Center for Chronic Disease Control authorized this study.

### Physical examination

All physical examinations were performed by trained medical staff. Physical examinations were mainly conducted to obtain the SBP and DBP, body mass index (BMI), waist circumference (WC) and hip circumference (HC). A standard mercury sphygmomanometer was used to measure participants' SBP and DBP after sitting for at least 5 minutes. WC and HC were measured using inextensible anthropometric tape with the participations standing straight, feet positioned close together and arms at the sides.

### Biochemical measurements

Biochemical measurements included glucose (Glu), total cholesterol (TC), triglycerides (TG), uric acid (UA), and plasma Hcy. The uricase method was used to quantitatively test the UA level. Enzymatic methods were adopted for the measurement of TC, Glu, and TG, and a circulating enzymatic method was employed to measure the Hcy level. All bioindicators were measured by an automatic biochemical analyzer (HITACH 7080, Tokyo, Japan).

### SYBR Green-based quantitative methylation-specific PCR (qMSP)

The specific steps in DNA extraction and bisulfite conversion were performed as previously described (22). The details of qMSP have also been described in a previous study (23). The *CBS* gene was amplified with the following primers: 5'-GGATGGAGTTATA TTATGAAGGT-3' for the forward primer and 5'-AACAATC TCGCTCAATCG-3' for the reverse primer. Simultaneously, 5'-GTGATGGAGGAGGTTTA GTAAGTT-3' and 5'-CCAATAAAACCTACTCCTCCCTTAA-3' were used as forward and reverse primers to amplify *ACTB*, respectively. The reaction was conducted under the following conditions: denaturation at 95°C for 600 sec, followed by 45 cycles at 95°C for 20 sec, annealing at 56°C for 45 sec and then 72°C for 20 sec. A melting curve step was performed at 95°C for 15 sec and 60 sec at 60°C, with a temperature rising at 0.11°C per second up to 95°C to measure the fluorescence signal. The percentage of methylated reference (PMR) of the *CBS* was used to quantitate *CBS* methylation.

### Statistical analysis

The mean±standard deviation (SD) and *t*-test/ANOVA were used to present and analyze continuous variables. Frequencies (percentages) and Chi-squared tests were used to express and analyze categorical variables. Odds ratios (ORs) and 95% confidence intervals (CIs) were calculated to estimate the risk of different factors in hypertension and stroke with logistic regression models. In stratified analysis, potential modifiers, such as sex (male or female), age (<65 or ≥65 years), BMI (<24 or ≥24 kg/m^2^), homocysteinemia (<15 or ≥15 μmol/l), smoking (yes/no), and drinking (yes/no), were assessed. The interaction between the PMR-CBS and stratified factors was examined after adjustment for age, sex, BMI, Hcy, UA, TG, TC, Glu, WC, HC, SBP, DBP, drinking history and smoking history. Pearson's correlation analyses was performed to evaluate the relation between *CBS* methylation level and age. We used the area under the curve (AUC) to evaluate the diagnostic value of *CBS* methylation for hypertension and stroke. All statistical analyses were conducted by SPSS version 18.0 (SPSS, Inc., Somers, NY, USA). A two-sided *p*<0.05 was significant.

## RESULTS

**Figure 1:**
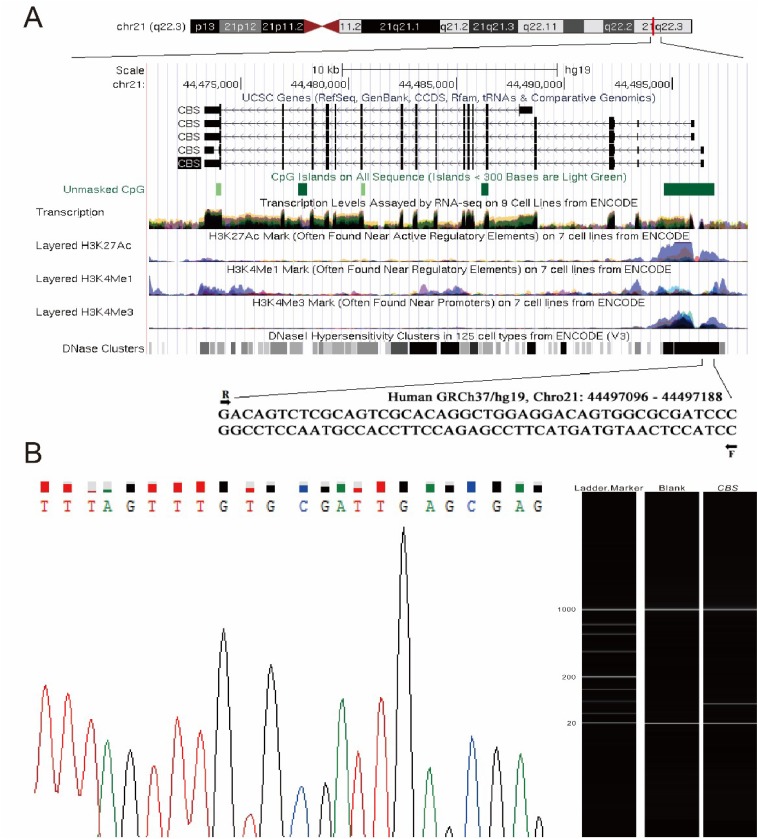
Methylation assay of the *CBS* gene and its quality control. (A) The abridged general view of five CpG sites on the *CBS* gene promoter. F: forward primer; R: reverse primer. The genomic positions and functional annotations of *CBS* were obtained from the UCSC genome browser according to human 2013 (GRCh37/hg19). (B) Methylation *status* of the *CBS* gene in patients with hypertension was analyzed by quantitative methylation-specific PCR.

A total of five CpG sites were selected from a fragment on the *CBS* promoter (Figure 1A). Bisulfite conversion by sequencing the converted DNA samples showed good results (Figure 1B). This case-control study recruited 132 stroke patients, 218 age- and gender-matched healthy individuals and 243 matched hypertensive patients.

**Figure 2:**
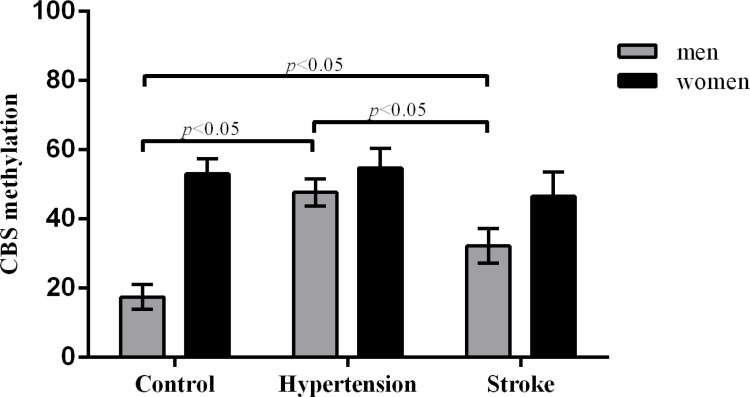
Comparison of *CBS* methylation levels between healthy controls and hypertensive and stroke patients.

Table 1 shows no significant differences in age, gender, UA, TG, TC, and smoking *status* of different groups, whereas the PMR level of *CBS* was higher in hypertension and stroke patients than in healthy controls (mean PMRs were 50.61%, 38.05% and 30.53%, respectively, *p*<0.001). The *CBS* promoter methylation levels of male subjects in the healthy control, hypertensive and stroke groups were significantly different (mean PMRs were 17.50%, 47.70%, and 32.18%, respectively, *p*<0.001, Figure 2), and those of female subjects were not significantly different (*p*=0.142). The CBS promoter methylation in males was lower than that in females (all *p*<0.001). The mean Hcy levels in the hypertension and stroke groups were 16.97 μmol/L and 21.13 μmol/L, respectively, higher than 14.57 μmol/L in the healthy control group (*p*<0.001). BMI, Glu, WC, HC, SBP and DBP in the hypertension and stroke groups were also higher than those in the healthy controls (all *p*<0.05). The status of antihypertensive drugs in stroke was also better than that in hypertension (*p*=0.008).

**Table 1:** Baseline characteristics of healthy controls, hypertensive patients, and stroke patients.

	Healthy controls	Hypertensive patients	Stroke patients	*F*/χ^2^	*p*
Age (y)	64.35 ± 9.22	66.19 ± 9.84	65.77 ± 9.32	2.28	0.103
Gender (M/F)	138/80	143/100	78/54	1.10	0.576
Hcy (μmol/L)	14.57 ± 5.70	16.97 ± 12.89	21.13 ± 18.56	11.31	<0.001
UA (μmol/L)	365.89 ± 97.06	364.50 ± 97.67	365.91 ± 87.25	0.02	0.985
TG (mmol/L)	1.75 ± 1.47	1.92 ± 1.63	1.79 ± 0.91	0.92	0.401
TC (mmol/L)	5.12 ± 1.02	5.11 ± 1.03	4.91 ± 1.07	2.00	0.137
BMI (kg/m^2^)	23.46 ± 3.08	24.44 ± 2.93*	24.49 ± 2.78*	7.82	0.001
Glu (mmol/L)	4.96 ± 0.81	5.67 ± 1.34*	5.64 ± 1.07*	27.33	<0.001
WC (cm)	84.69 ± 9.80	87.03 ± 9.00*	87.11 ± 10.38*	4.18	0.016
HC (cm)	93.62 ± 9.54	95.69 ± 7.99*	95.15 ± 9.63	3.22	0.041
SBP (mmHg)	123.57 ± 13.81	134.43 ± 16.47*	135.69 ± 15.86*	37.40	<0.001
DBP (mmHg)	77.95 ± 8.43	82.59 ± 11.18*	82.76 ± 11.27*	14.41	<0.001
Smoking (no/yes)	182/36	214/29	115/17	2.15	0.341
Drinking (no/yes)	136/82	176/67	112/20	20.53	<0.001
Antihypertensive drugs (no/yes)	–	50/193	13/119#	7.04	0.008
PMR-*CBS* (%)	30.53 ± 26.91	50.61 ± 25.73*	38.05 ± 24.66* #	34.93	<0.001

Hcy: plasma homocysteine, UA: uric acid, TG: triglycerides, TC: total cholesterol, BMI: body mass index, Glu: blood glucose, WC: waist circumference, HC: hip circumference, SBP: systolic blood pressure, DBP: diastolic blood pressure, PMR: percent of methylated reference.

* *p*<0.05, compared with healthy controls.

# *p*<0.05, compared with hypertensive patients.

As shown in Table 2, multivariable logistic regression analysis indicated that *CBS* promoter hypermethylation significantly increased the risk of hypertension and stroke, and the ORs (95% CI) were 1.035 (1.025–1.045) and 1.015 (1.003–1.028), respectively. In addition, Hcy was related to the development of hypertension and stroke, and the ORs (95% CI) were 1.040 (1.004–1.084) and 1.081 (1.035–1.129), respectively.

**Table 2:** Association of CBS methylation and the risk of hypertension and stroke adjusted by multivariable logistic regression models.

	Hypertension		Stroke	
Variable	OR	95% CI	OR	95% CI
CBS methylation	1.035	1.025-1.045	1.015	1.003-1.028
Gender (F/M)	0.840	0.454-1.554	1.049	0.500-2.200
Age (y)	1.001	0.972-1.030	0.995	0.963-1.028
Hcy (μmol/L)	1.040	1.004-1.084	1.081	1.035-1.129
UA (μmol/L)	0.999	0.997-1.002	1.000	0.996-1.003
TG (mmol/L)	0.902	0.739-1.102	0.830	0.603-1.142
TC (mmol/L)	0.969	0.746-1.259	0.760	0.565-1.021
BMI (kg/m^2^)	1.071	0.964-1.190	0.969	0.929-1.011
WC (cm)	0.972	0.935-1.011	0.969	0.929-1.011
HC (cm)	1.034	1.003-1.066	1.017	0.980-1.055
SBP (mmHg)	1.051	1.032-1.071	1.051	1.026-1.077
DBP (mmHg)	1.039	1.014-1.065	1.017	0.984-1.051
Glu (mmol/L)	2.227	1.651-3.004	2.233	1.597-3.122
Drinking (yes/no)	0.708	0.499-1.003	0.369	0.220-0.618
Smoking (yes/no)	1.204	0.555-2.612	1.073	0.427-2.693

Hcy: plasma homocysteine, UA: uric acid, TG: triglycerides, TC: total cholesterol, BMI: body mass index, Glu: blood glucose, WC: waist circumference, HC: hip circumference, SBP: systolic blood pressure, DBP: diastolic blood pressure, PMR: percent of methylated reference, OR: odds ratios, 95% CI: 95% confidence intervals.

**Figure 3:**
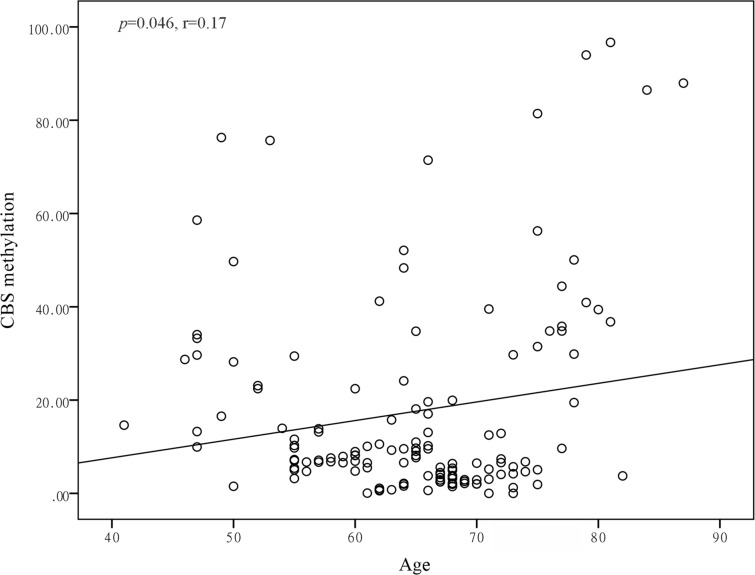
Pearson correlation between age and CBS methylation in male healthy controls.

Potential effect modification of the association of CBS promoter methylation with hypertension and stroke by age, gender, BMI, Hcy, drinking and smoking history were examined in subgroups analyses, while significant effect modification was observed by gender in patients with hypertension (*p*-values for interaction<0.001) and gender in patients with stroke (*p*-value for interaction=0.035, Table 3). *CBS* promoter hypermethylation increased the risk of male hypertensive patients (OR=1.057; 95% CI = 1.041–1.073), male stroke patients (OR: 1.028; 95% CI: 1.011–1.044), female hypertensive patients (OR: 1.011; 95% CI: 0.996–1.026) and female stroke patients (OR: 0.992; 95% CI: 0.970–1.015). Subsequently, we performed correlation tests between PMR levels of *CBS* and clinical variables in healthy subjects, and the results showed a positive correlation between the PMR level of the *CBS* promoter and age (*r*=0.17, *p*=0.046, Figure 3) in healthy male controls.

**Table 3:** Multivariate-adjusted odds ratios and 95% confidence intervals of hypertension and stroke for CBS methylation in subgroups.

		Hypertension		Stroke	
		OR (95% CI)*	*p*-interaction	OR (95% CI)*	*p*-interaction
Gender			<0.001		0.007
	Men	1.057 (1.041-1.073)		1.028 (1.011-1.044)	
	Women	1.011 (0.996-1.026)		0.992 (0.970-1.015)	
Age			0.635		0.836
	<65 y	1.037 (1.019-1.056)		1.013 (0.989-1.037)	
	≥65 y	1.038 (1.025-1.052)		1.021 (1.005-1.037)	
BMI			0.325		0.951
	<24 kg/m^2^	1.038 (1.023-1.054)		1.014 (0.996-1.032)	
	≥24 kg/m^2^	1.037 (1.023-1.052)		1.019 (1.000-1.038)	
Homocysteinemia		0.191		0.932
	<15 μmol/L	1.032 (1.019-1.044)		1.014 (0.997-1.031)	
	≥15 μmol/L	1.050 (1.031-1.071)		1.020 (0.999-1.042)	
Drinking			0.761		0.459
	No	1.031 (1.020-1.042)		1.016 (1.002-1.029)	
	Yes	1.060 (1.033-1.087)		1.015 (0.981-1.051)	
Smoking			0.861		0.794
	No	1.036 (1.025-1.047)		1.014 (1.001-1.027)	
	Yes	1.077 (1.026-1.131)		0.945 (0.854-1.044)	

OR: odds ratios, 95% CI: 95% confidence intervals.

* Adjusted for age, sex, BMI, homocysteine, uric acid, triglycerides, total cholesterol, plasma glucose, waist circumference, hip circumference, systolic blood pressure, diastolic blood pressure, drinking and smoking.

**Figure 4:**
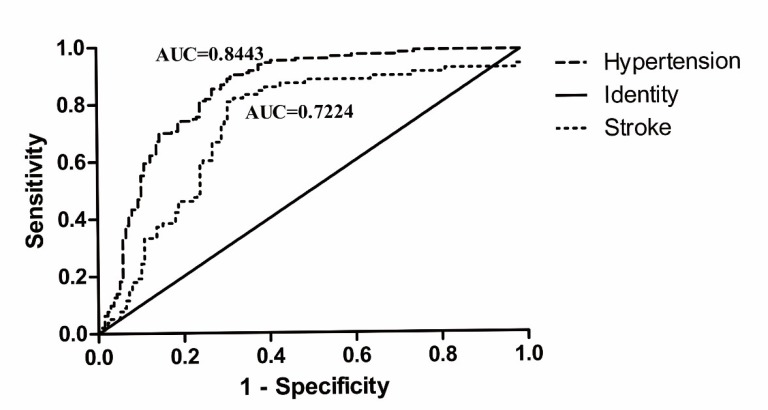
Receiver operating characteristic (ROC) curve of CBS methylation in male hypertensive and stroke patients.

ROC curves were generated to evaluate the role of the PMR level of *CBS* in the diagnostic value of hypertension and stroke. The AUC of *CBS* promoter methylation was 0.844 (95% CI, 0.796–0.892) in male hypertensive patients and 0.722 (95% CI, 0.653–0.799) in male stroke patients (Figure 4).

## DISCUSSION

The purpose of the present matched study was to examine the risk of abnormal *CBS* methylation in hypertensive and stroke patients. The results revealed that *CBS* promoter hypermethylation was an independent risk factor for hypertension and stroke, especially in men. ROC curve analysis also suggested that *CBS* promoter hypermethylation was a potential biomarker in male subjects for the diagnosis of hypertension and stroke.

Accumulating evidence, as well as our findings, indicates that Hcy may be related to hypertension and stroke (24). The *CBS* enzyme, one of three predominant enzymes, is involved in the process of Hcy conversion to cysteine and contributes nearly 40–50% of cysteine production (25). *CBS* deficiency was identified as a genetic factor that resulted in elevated levels of Hcy or hyperhomocysteinemia (25). Hypermethylation levels of the *CBS* gene were observed in hypertension and stroke patients, which might lead to silencing of *CBS* gene expression and accumulation of Hcy levels, thus contributing to the susceptibility to hypertension and stroke. In addition, the PMR level of *CBS* was lower in stroke patients than hypertensive patients. In acute ischemic stroke conditions, *CBS* expression was activated and increased, causing increased production of H2S (26,27). *CBS* gene transfection of SH-SY5Y cells would results in production of enzymes to synthesize H2S, which would enhance cell death and exacerbate ischemic injuries (26).

The difference in *CBS* methylation levels was statistically significant only in males, and gender differences were observed in several genes (28, 29). Renal *CBS* enzyme levels in C57BL/6J mice were higher in males than females (30). Males and females had different susceptibilities to hypertension, and males were more susceptible (31). Sexual dimorphism in mammalian gene expression is thought to result in different sex hormone (testosterone, androgens) levels in males and females (32). Testosterone has a distinct impact on renal *CBS* enzyme levels, and when female mice were injected with androgen, the renal *CBS* activity increased twofold (33). Androgens mainly regulate renal *CBS* levels by complex organ-specific regulation (30).

Additionally, age had a positive correlation with promoter hypermethylation (34), and methylation levels in several genes were shown to increase with aging (34,35). Our study also demonstrated a similar association between age and CBS methylation in males. However, we adjusted for age by multivariable logistic regression analysis and confirmed that the PMR level of *CBS* was an independent risk factor both in hypertension and stroke.

To the best of our knowledge, the present work is the first study with a large samples size to assess the influence of *CBS* methylation on hypertension and stroke. In addition, as this is a matched case-control study, the influence of age on methylation levels was controlled. However, the following limitations should be noted. First, the genetic ancestry link is different in different ethnic groups and has a major impact on the DNA methylation level, leading to differences in individual risk to diseases (36,37). Because the subjects enrolled in this study were only Chinese Han individuals from Shenzhen, our conclusion might not be applicable to other ethnic groups. Second, only the PMR level of the *CBS* gene was measured, and DNA methylation of other genes involved in Hcy metabolism may also confer susceptibility to hypertension and stroke. Third, we did not perform gene expression analysis, and the conclusions of our study are not causal. Finally, although we adjusted for confounding factors that may affect the PMR level of *CBS*, potential confounding factors such as medical treatment (38) may have an effect.

In conclusion, male patients with hypertension and stroke had increased *CBS* methylation levels. In Chinese Han males, *CBS* hypermethylation in the blood might serve as an independent biomarker for the diagnosis of hypertension and stroke. Further large-scale studies in different races with gene expression analyses should be performed to verify our findings.
